# A New Model Employing Fermi-Level Integration Limits to Correct Systematic Errors in the Extraction of Semimetal Carrier Densities and Mobilities

**DOI:** 10.1021/acs.nanolett.6c02845

**Published:** 2026-08-21

**Authors:** Y. W. Sun, D. Holec, O. Fenwick, M. J. Coak, D. G. Papageorgiou, C. J. Humphreys, D. J. Dunstan

**Affiliations:** † School of Physics and Astronomy, 1724 University of Birmingham, Birmingham B15 2TT, United Kingdom; ‡ School of Engineering and Materials Science, 4617 Queen Mary University of London, London E1 4NS, United Kingdom; ¶ Department of Materials Science, Montanuniversität Leoben, Leoben 8700, Austria; § School of Physical and Chemical Sciences, 4617 Queen Mary University of London, London E1 4NS, United Kingdom

**Keywords:** graphene, carrier density, Hall effect, mobility, semimetals, Fermi level

## Abstract

We demonstrate that the accepted model for transport in semimetals gives incorrect results and introduce a corrected scheme that reproduces experimental data. Carrier densities in semimetals have been systematically overestimated by factors of 2–5 throughout the literature, leading to underestimated mobilities by the same factor. This resolves the long-standing puzzle of why nominally identical graphene samples show order-of-magnitude mobility variations. By integrating the density of states from the Fermi level rather than from band edges, we reproduce the full evolution of the Hall coefficient and longitudinal resistance across five independent graphene data sets with zero fitting parameters. A double-normalized polar representation reveals a decisive signature: a smooth arc that experimental data match, while no realistic density of states or mobility rescues the band-edge model. Our Fermi model provides a parameter-free, temperature-robust description with immediate implications for reevaluating mobility benchmarks across the 2D materials literature.

## Graphical abstract

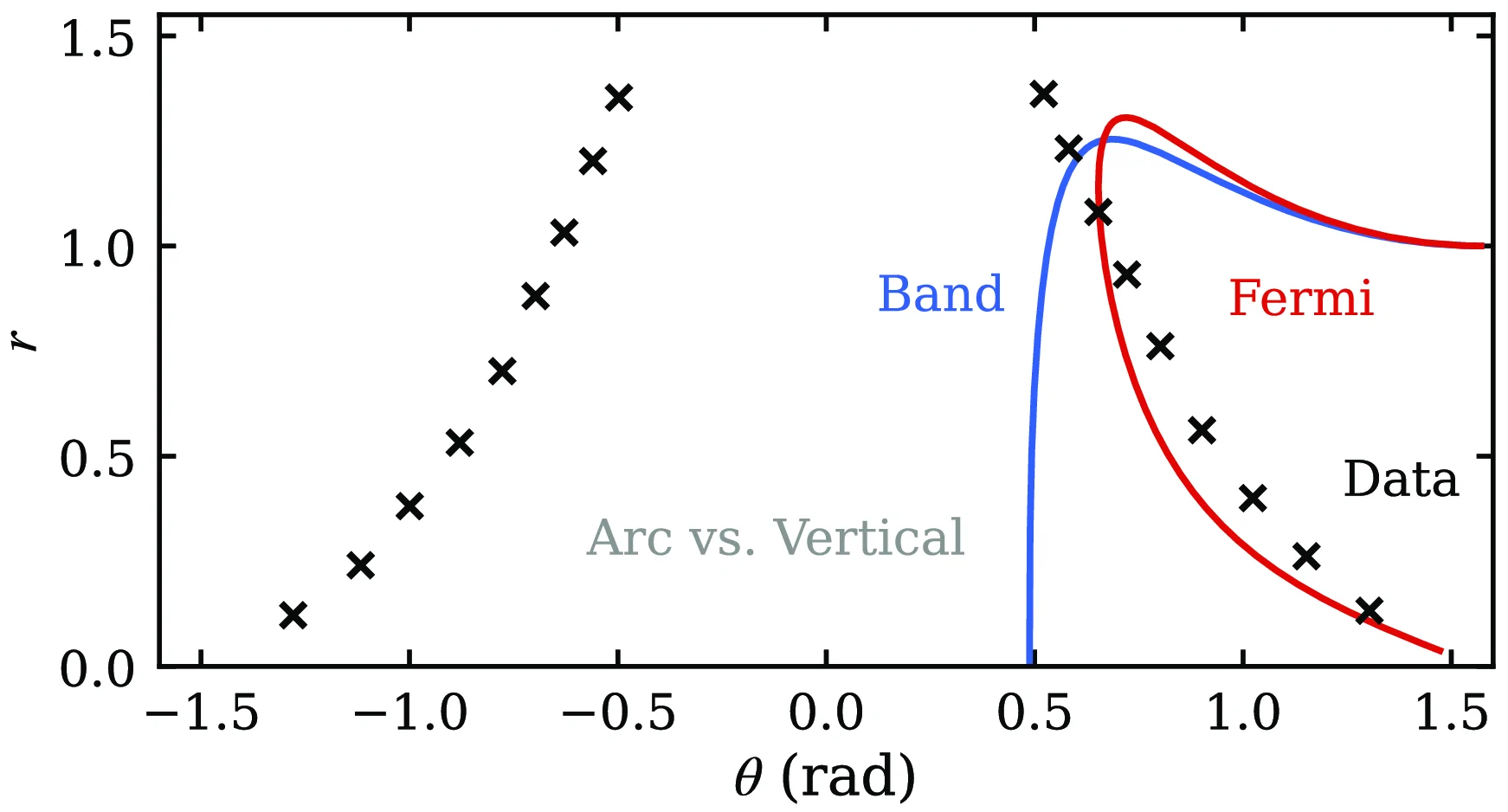

The carrier density and mobility are key parameters for characterizing the electrical properties of materials. They are not directly measurable but are typically inferred from combined Hall effect and resistance measurements.^1^ The carrier density is calculated as *n* = 1/(*eR*_H_), where *R*_H_ is the Hall coefficient and *e* is the elementary charge, and the drift mobility follows from the measured resistivity, in sheet geometry μ_s_ = 1/(*enR*_*xx*_). In materials such as lightly doped semiconductors and semimetals, both electrons and holes contribute to the Hall coefficient and resistance: $R_{H}=\frac{p{\mu_{p}}^{2}-n{\mu_{n}}^{2}}{e{\left(p\mu_{p}+n\mu_{n}\right)}^{2}}$ and $R=\frac{l}{e\left(n\mu_{n}+p\mu_{p}\right)A}$ , where *n* is the electron density, *p* is the hole density, μ_n_ is the electron mobility, and μ_p_ is the hole mobility.^2^ Extracting the four parameters *n*, *p*, μ_n_, and μ_p_ from two measurements requires an established relationship among them, independent from the measurement methods themselves. A fundamental consideration in transport is that electrons inside the Fermi surface in *k*-space contribute a negative Hall voltage, while the empty states inside contribute a positive Hall voltage. In the conventional semiconductor picture, the latter are represented by holes (empty states in an otherwise filled valence band). The extraction is often simplified by assuming conduction is dominated by a single carrier type, with well-known limitations.^3,4^

A more accurate two-carrier model invokes electron and hole densities. The carrier densities at a given Fermi level and temperature are calculated by integrating the product of the material’s density of states (DoS) and the Fermi–Dirac distribution (F–D) over the band: $n=\int_{E_{C}}^{\infty }D\left(E\right)\;f\left(E\right)$ and $p=\int_{-\infty }^{E_{V}}D\left(E\right)\;\left[1-f\left(E\right)\right]$ , where *E*_C_ is the conduction band edge and *E*_V_ is the valence band edge.^5^ In this Letter, we examine this relationship by comparing results calculated using the conventional band-edge method with experimental data from simultaneous Hall coefficient and sheet resistance measurements on graphene across various doping levels. Our investigation leads to a refined method. We change both integral limits *E*_C_ and *E*_V_ to the Fermi level, *E*_F_, $n=\int_{E_{F}}^{\infty }D\left(E\right)\;f\left(E\right)$ and $p=\int_{-\infty }^{E_{F}}D\left(E\right)\;\left[1-f\left(E\right)\right]$ , and the results of this Fermi method align more closely with previously published experimental data.^6^ This change in the method of calculation corresponds to the physical Fermi model in which, in n-type semimetals, delocalized states of energy *E*, *E*_C_ < *E* < *E*_F_ give a positive Hall effect. Similarly, in p-type semimetals, delocalized states of energy *E*, *E*_F_ < *E* < *E*_V_ give a negative Hall effect. The improved agreement with experiment supports the Fermi model and the physical picture behind it.

It is important to emphasize that we are not proposing a new method to compute conductivity *ab initio*—textbooks correctly show that dc conductivity is determined by electrons near the Fermi level.^7^ Rather, we are correcting the standard extraction procedure used throughout the 2D materials community: experimentalists measure *R*_H_ and *R*_*xx*_, calculate carrier density using formulas derived from band-edge integration, and derive mobility from μ = σ/(*ne*) as mentioned above. In semimetals such as graphene, this procedure counts electrons that cannot respond to external fields (those deep in the band, far from *E*_F_), artificially inflating the carrier density and deflating the inferred mobility.

Consider graphene with equal electron and hole mobility. For a monotonic sweep of *E*_F_ (e.g., via gate voltage), the Hall coefficient rises from a small finite value at heavy *p* doping, peaks, crosses *R*_H_ = 0 at neutrality when *n* = *p*, and mirrors this evolution on the *n* side. The sheet resistance peaks at neutrality and falls symmetrically on both sides. As shown below, the quantitative relationship between these well-established trends^8,9^ exposes the inaccuracy of the band-edge method: carrier densities are systematically overestimated, so extracted mobilities are underestimated by the same factor, suggesting intrinsic mobilities to be significantly higher than currently believed.^10,11^

To generate theoretical predictions for comparison, we first calculate the Hall coefficient and sheet resistance from dual-carrier contributions as functions of the doping level, represented mathematically as $R_{H}=\frac{p-n}{e{\left(p+n\right)}^{2}}$ and ${\mathrm{R̅}}_{xx}=\frac{1}{e\mu \left(p+n\right)}$ . We employ a linear approximation for the DoS based on density functional theory (DFT) computations detailed previously (Figure S1),^12^ to calculate *n* and *p* over a sweep of *E*_F_ from −2.4 to 3.6 eV, every 5 meV, at 300 K. We employ two contrasting methods: the conventional “band model”, where integration covers the entire bands, and our proposed “Fermi model”, which integrates from the Fermi level to positive and negative infinity for *n* and *p* (with the corresponding F–D functions), respectively. In graphene, there is no physical reason for the band edge (Dirac point) to serve as the integral limit: the states just below and above the Dirac point are both π states derived from C p_*z*_ orbitals, with identical orbital character, angular momentum, and spin. An electron of energy just below the Dirac point is physically indistinguishable from one just above, yet band-edge integration treats them entirely differently. This contrasts with traditional semiconductors (e.g., GaAs), where conduction and valence bands have different orbital characters (s-like vs p-like), giving the band edge genuine physical significance as an integration boundary. This latter approach recognizes that transport is fundamentally driven by the delocalized states nearest to the Fermi level because only these can change the occupancy under external fields. Initially, we disregard doping-dependent mobility variations for simplicity, and we revisit their potential influence later. The relationship of these Fermi-level-referenced densities to Boltzmann transport theory—an integration-by-parts identity expressing them as transport-kernel-weighted state counts, their exact reduction to band-edge integration whenever *E*_F_ lies in a gap, and their finite zero-temperature limits—is derived in SI Section 6.

To clarify and amplify the differences between the band model and our proposed Fermi model, we introduce a double-normalized polar representation. After internally normalizing the Hall coefficient and sheet resistance by dividing each data set by its own maximum (thus isolating the intrinsic shape dictated by the carrier balance and DoS), we map these normalized quantities onto polar coordinates defined by

1 $$\theta =\arctan\left({\mathrm{R̃}}_{xx}/{\mathrm{R̃}}_{H}\right),,r=\sqrt{{{\mathrm{R̃}}_{H}}^{2}+{{\mathrm{R̃}}_{xx}}^{2}}$$

In this representation (Figure 1), θ reflects the balance between the Hall coefficient and sheet resistance and *r* how close each state is to maximum response. Because we normalize *R*_H_ and *R*_*xx*_ *independently* by their own maxima, (θ, *r*) is dimensionless and therefore independent of the absolute mobility scales. Notably, this normalization also renders the representation independent of the Hall scattering factor *s* = μ_H_/μ_d_: any constant multiplicative factor in *R*_H_ cancels upon normalization, ensuring that the trajectory shape reflects the underlying carrier physics rather than scattering-mechanism-dependent corrections.^13,14^ The trajectory shape thus provides a parameter-free experimental test: the band model predicts near-vertical high-doping trajectories (minority carrier exponentially suppressed), while the Fermi model predicts smooth arcs (both carriers persist near *E*_F_)—a qualitative distinction that no choice of absolute carrier density or mobility can reconcile. The contrast with conventional practice bears emphasis: used as an extraction tool, the band model converts measured curves into densities and mobilities through per-device free quantities—the absolute mobility, its doping and temperature dependences, and *s*—that exceed the number of measured curves, so agreement is guaranteed and tests nothing. The normalization removes exactly these quantities, and the comparison of Figure 1 therefore confronts both models with experiment with no fitting parameter on either side.

**Figure 1:**
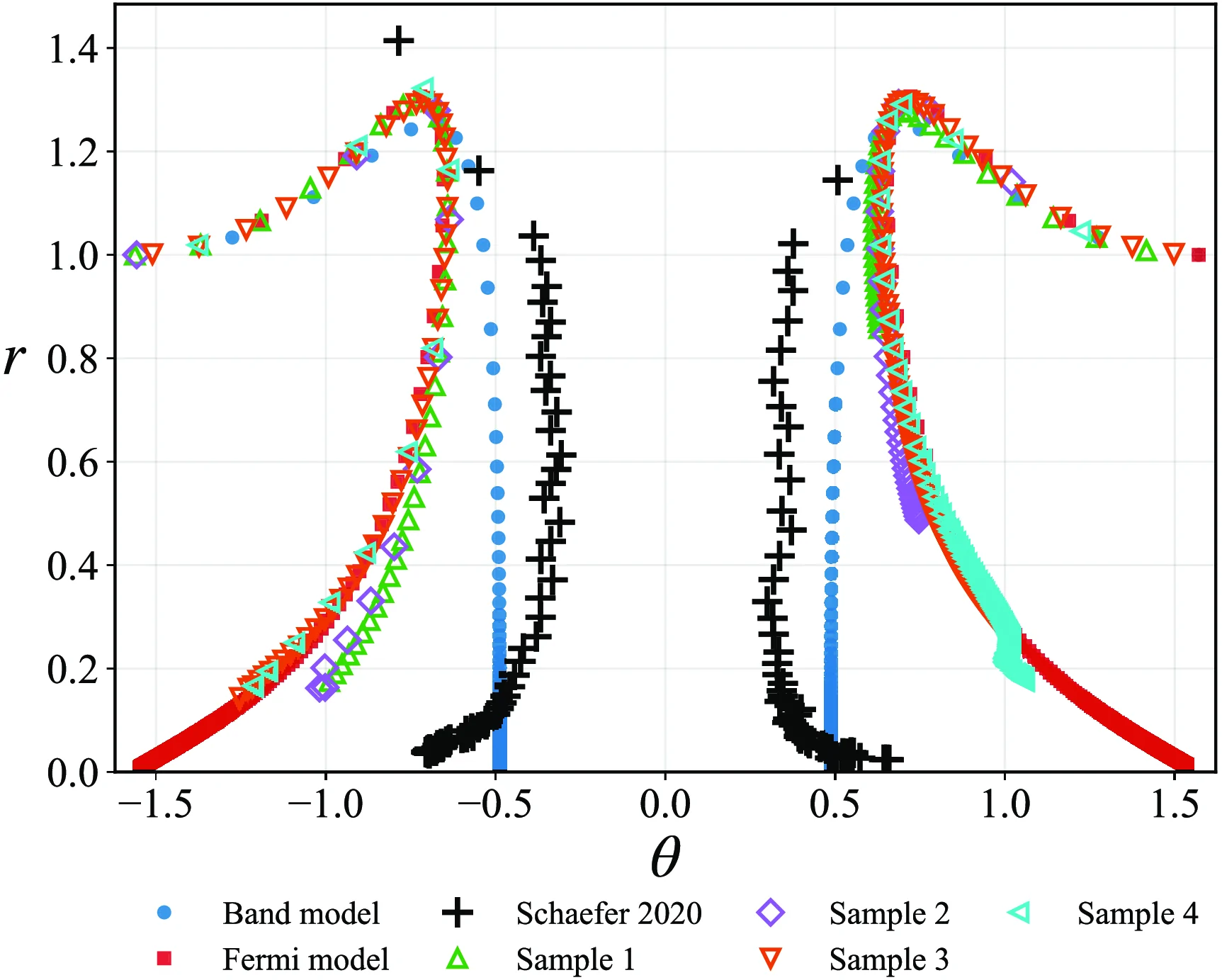
Double-normalized polar plot comparing theoretical predictions from the conventional band model and the proposed Fermi model with five experimental data sets: published h-BN/graphene data from Schaefer et al.^6^ (black crosses) and four graphene-on-sapphire samples (Samples 1–4, open markers). Each curve traces the evolution of the normalized Hall coefficient and sheet resistance as doping changes monotonically from strongly p-type (left side) through neutrality (θ = ±π/2, *r* = 1) to strongly n-type (right side). The band model (blue circles) exhibits nearly vertical trends with increasing doping, while the Fermi model (red squares) shows smoother, wider arcs. All four sapphire samples collapse onto the gapless Fermi-model arc with no free parameters; the Schaefer data set is laterally shifted, consistent with a small band gap discussed in Figure 2.

Critically, when published h-BN/graphene data from Schaefer et al.^6^ and four further graphene-on-sapphire samples are recast into our double-normalized polar coordinates, all five data sets exhibit arcs consistent with the Fermi model. The four sapphire samples coincide with the gapless Fermi-model curve; the Schaefer data set is laterally shifted but follows the same arc shape, discussed below in the context of substrate-induced band gaps. As *r* decreases toward zero with high doping, there is an arc toward high θ, in stark contrast to the band model where the line is almost vertical at θ ≈ ±0.5. The key distinction is the trajectory shape, not the absolute position: experimental data follow the Fermi model’s gradual arc, while the band model shoots vertically once |*E*_F_| exceeds ≈50 meV—a criterion independent of any particular definition of mobility or carrier density. With constant mobility (the simplest assumption), the Fermi model reproduces the experimental trajectory with zero free parameters. The band model, by contrast, would require a finely tuned doping-dependent mobility function μ­(*E*_F_) to produce a similar arc—and such a function would need to be universal across samples with vastly different defect levels, grain sizes, and doping mechanisms, an implausible coincidence.

So far, our calculations have used an ideal linear DoS. Real samples, however, can exhibit (i) steeper or shallower DoS slopes, (ii) residual states (forming a nonzero valley), or (iii) a small band gap (e.g., induced in h-BN/graphene heterostructures).^15^ Figure 2 compares how such DoS modifications affect the band and Fermi models in polar space.

**Figure 2:**
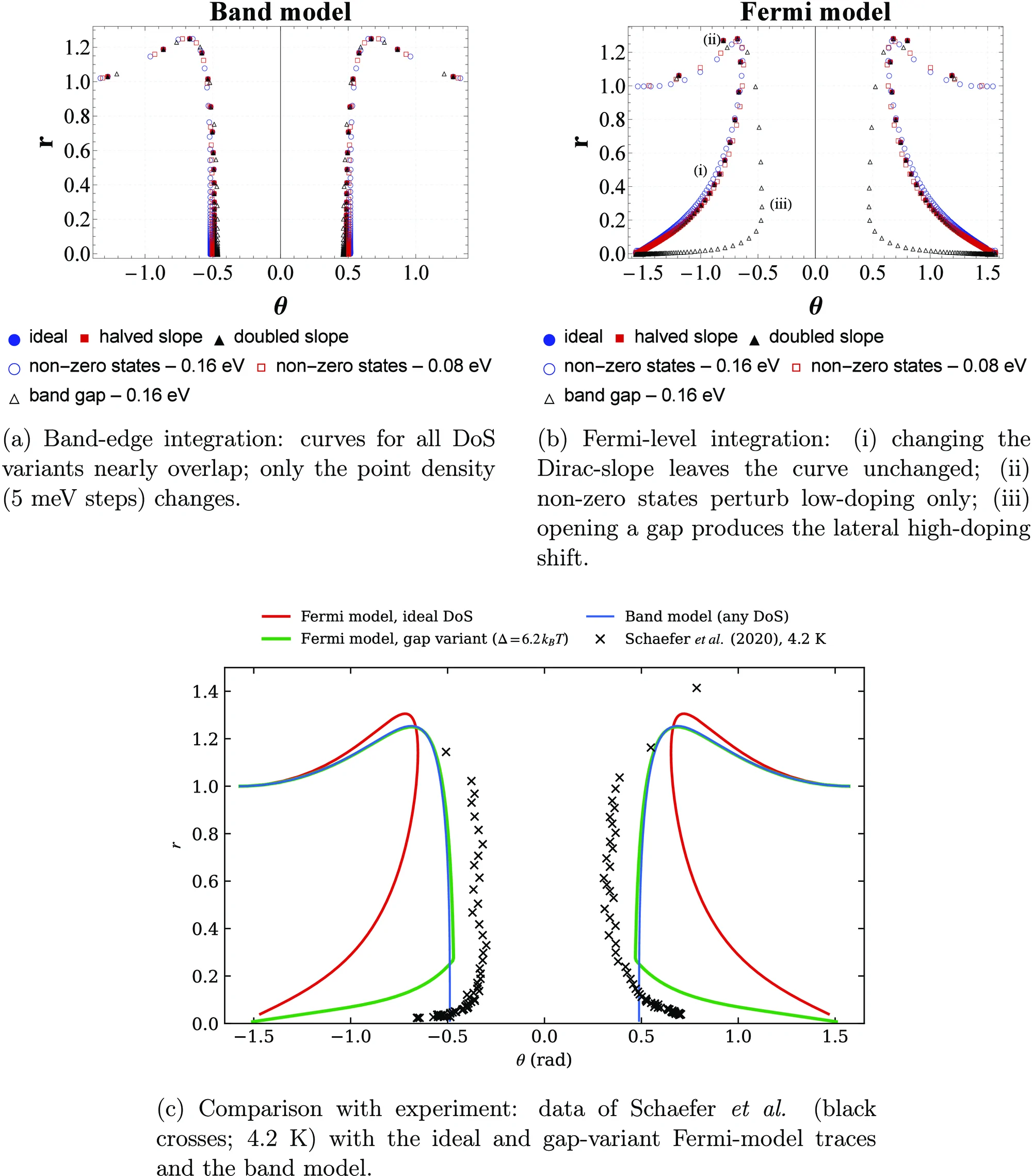
(a and b) Normalized polar comparison of band-edge vs Fermi-level counting across DoS variants. Panel c compares the models with the data of Schaefer et al.^6^ (black crosses; 4.2 K); the plotted gap variant has the dimensionless gap Δ/*k*_B_ *T* = 6.2, the only combination through which a gap enters these coordinates (see the text). While *E*_F_ lies within the gap, the Fermi prescription reduces exactly to band-edge integration, so the gap-variant trace follows the band-model line and departs from it at the band edge—the visible turning point (SI Section 6).

For the band model (Figure 2a), all DoS variants collapse onto a single rigid trace. The Fermi model (Figure 2b) is inherently DoS-sensitive: slope changes leave the trace unchanged, a narrow valley of residual states perturbs it only near maximum *r*, while a gap shifts the high-doping segment laterally before the trace curls toward (θ → π/2, *r* → 0).

Introducing a band gap on the order of tens of millielectronvolts (typical for near-aligned Gr/h-BN) as a single parameter laterally shifts the Fermi-model arc and can improve alignment with experiment; larger gaps up to 160 meV have been predicted for graphene sandwiched between h-BN layers or with additional fields.^6,15^ Because the normalized trajectory of the ideal DoS is temperature-invariant (Figure 4), a gap enters the representation only through the ratio of Δ to the energy window *E*_w_ over which the occupation of states is smeared, and single-parameter estimates place the Schaefer trajectory at Δ ≈ 6–9*E*_w_ (Figure 2c). At the 4.2 K of that measurement,^6^ *E*_w_ is set by the reported charge inhomogeneity of the device (δ*n* ≈ 2–4 × 10^9^ cm^–2^, i.e., ≈5–7 meV) rather than by *k*_B_ *T*, giving Δ of order 30–60 meV—the scale of gaps reported for aligned graphene/h-BN and predicted (≈50 meV) for commensurate stacking^15^—with a conservative, purely thermal lower bound of ≈2 meV. Residual differences from reported structural gaps are expected: the magnitude and functional form of the suppression (a hard gap versus a rounded suppression or residual in-gap states, cf. variants 4–6) are conditional on the assumed DoS family, and the low-temperature smearing window is disorder—rather than temperature—set. The rigorous treatment of this inverse problem—reconstructing *D*(*E*) from measured (*R*_H_, *R*), with its parametrization degeneracies near neutrality, data-weighting conventions, and model-comparison requirements—is a substantial methodology in its own right and the subject of dedicated forthcoming work. No analogous rescue is possible within the band-edge framework, providing compelling evidence that carrier densities in graphene should be obtained by integration from the Fermi level rather than over the full band. Thus, Schaefer’s data set serves as an external benchmark that only a DoS-sensitive, Fermi-level-referenced scheme can meet.

We mentioned that, in graphene, there is no physical reason for the band edge to serve as the integral limit. Wallace’s seminal π-band calculation already implied that *E*_F_, rather than the band extrema, is the natural boundary for counting carriers in graphite-type semimetals.^16^ This principle extends beyond graphene: the band-edge model’s success in traditional semiconductors stems from their highly asymmetric band structures (s-like conduction bands versus p-like valence bands), as well as their large band gaps, making the convenient mathematical approximation adequate despite being physically incomplete. For graphene, modern two-band analyses, nonetheless, integrate from fixed edges and then add four fitting parameters (*n*, *p*, μ_e_, μ_h_) to match *R*_H_(*V*_g_) and σ­(*V*_g_).^18^ That procedure works because of the low doping range of the data, where the experimental uncertainty exceeds the difference between the band and Fermi model predictions, and of the extra tunability in mobilities. In contrast, our Fermi-integration scheme turns a four-parameter fit into a zero-parameter prediction, giving a rising θ at low *r* that is found experimentally where high doping data are available.^6^

So far we take the simplest case μ_n_ = μ_p_ = μ_0_, independent of the carrier density. In practice, two effects enter: (a) the mobility of both carriers falls by a factor $f=\frac{\mu_{0}}{\mu_{n}}≃1.5$ at high doping in typical graphene devices,^19^ and (b) electron and hole mobilities may differ slightly, μ_p_ = βμ_n_ with β ≈ 1.1–1.3 in h-BN/graphene heterostructures. At high doping, the two carrier-counting schemes respond in different ways. In the band-edge model, the minority carrier is suppressed, so ${\mathrm{R̃}}_{H}\propto 1/n$ , while ${\mathrm{R̃}}_{xx}\propto f/n$ ; hence, a factor of *f* = 1.5 shifts the band model curve in Figure 1, from θ_*∞*_ ≃ 0.5 rad to arctan­[*f* tan 0.5] = 0.65 rad, matching the experimental data at high doping but leaving the data points of θ ≲ 0.5 at the medium doping range unexplained. A modest asymmetry μ_p_/μ_n_ ≠ 1 hardly affects the band model curve at high doping (the unequal mobility is multiplied by an exponentially small minority density). Both density-dependent mobility and electron–hole asymmetry grant the Fermi model more (but perhaps not necessary) tunability to match the experimental data. Therefore, including realistic mobility effects strengthens rather than weakens the argument for counting carriers from the Fermi level. This robustness is geometric: while these effects shift trajectory angles and introduce n–p asymmetry, they cannot transform a vertical line into an arc—the qualitative shape distinction persists regardless of mobility parameters.

We will now discuss the implications and the associated new understandings of the Fermi model. While certain points below may initially appear to be surprising, they are, upon closer examination, entirely reasonable. Before discussing our results, we clarify our terminology: throughout this work, “electrons” refers to occupied states contributing a negative Hall effect (those outside the Fermi surface in *k*-space, under the Fermi model), while “holes” refers to empty states contributing a positive Hall effect (vacancies inside the Fermi surface).

- **The electron density increases with p-type doping and so does the hole density with n-type doping.** This is a consequence of the increasing DoS near the Fermi level with both p- or n-type doping. We note that, in the case of n-type doping (as an example), the electron density consistently exceeds the hole density, in a linear or parabolic DoS. This occurs because integration for the electron density is effectively conducted over half the F–D above the Fermi level, where the DoS is invariably greater than that in the corresponding half-width below, over which the integration is for the holes. Figure 3a plots the Fermi-model electron density, the sum, and the difference of the two densities against Fermi-level shift for an ideal DoS. In scenarios with a linear DoS, such as this, the difference between electron and hole densities stabilizes once the Fermi level surpasses half the F–D width from the Dirac point. This constant difference is at a magnitude significantly lower than the densities of electrons and holes themselves, suggesting a persistent dual-carrier contribution throughout the entire doping range. We emphasize that the total induced charge—the quantity fixed by gate electrostatics and counted by quantized Hall plateaus—is the same occupation integral over all states in both schemes and is left untouched by our correction; the schemes differ only in the partition of the zero-field response into transport-active electrons and holes (SI Section 6). Figure 3b shows the familiar band-model counterpart. The practical consequence is striking: at typical doping levels (|*E*_F_| ∼ 0.1 eV), the Fermi model yields carrier densities ∼3 times lower than the band model (Figure 3). This directly implies that all published graphene mobilities based on Hall measurements are underestimated by a factor of ∼3.
- **The carrier densities calculated from the Fermi model are 2–5 times (for*E*_F_= 73–196 meV) lower than those from the band model, as is evident from a comparison of parts a and b of Figure 3, directly implying that all transport mobilities reported for graphene (and other semimetals) are systematically underestimated by the same factor.** This resolves the long-standing puzzle of why nominally identical graphene samples show mobility variations spanning an order of magnitude; these variations largely reflect the doping level at which measurements were performed—itself influenced by atmospheric doping, the rate of which varies with the relative humidity—not intrinsic sample quality differences. The Fermi model’s accounting for carriers solely within half the F–D from the Fermi level naturally leads to this correction. For a Fermi circle, the field-induced current can be described either as all electrons drifting collectively at centimeters per second—the picture underlying band-edge integration—or, equivalently and more physically, as only the electrons within *k*_B_ *T* of the Fermi surface responding, each at kilometer-per-second speeds; the Fermi model adopts the latter. The equivalence holds only for isotropic Fermi surfaces: for the anisotropic surfaces of real semimetals, the uniform-drift picture fails, and the Fermi model’s advantage is physical (correctly handling velocity anisotropy), not merely numerical (SI Section 4). This distinction explains why cyclotron-resonance values cannot be straightforwardly compared to values extracted via band-edge integration—the two methods implicitly define “carriers” differently. Despite the significant differences in the carrier density and its dependence on doping between the band and Fermi models (Figure 3), both approaches make comparable predictions for the Hall coefficient’s behavior with doping (Figure S3). This is notable because Hall effect measurements, which effectively distinguish between electron and hole contributions, are not sufficient on their own to differentiate between these two distinct models. Only through a detailed quantitative analysis combined with resistance measurements, such as the one presented here, can the models be accurately contrasted and the Fermi model’s validity be demonstrated.
- **Temperature variations leave the polar trace shape invariant.** One might expect the polar curves to shift as the F–D width doubles from 200 to 400 K; in the double-normalized coordinates, they do not. Figure 4 shows the polar traces for the band and Fermi models at 200, 300, and 400 K (ideal DoS and constant mobilities). All three temperatures collapse on a single curve; temperature merely rescales the rate of doping (the intervals between the neighboring points are fixed at 5 meV). Therefore, the polar trace holds even when the temperature shifts the Fermi level.
- **The Fermi model provides a universally applicable and quantitatively straightforward description of electrical conductance and the Hall effect.** Fundamentally, the electrical conductance of a material is driven by the drift of the Fermi surface in response to an external field, as illustrated in Figure S4.^20^ This drift is characterized by the field-induced asymmetry of occupied (and unoccupied) states relative to the origin in *k*-space, representing electrons (and holes). Crucially, such asymmetry occurs not just at the band edges but also near the Fermi level, leading to the simultaneous presence of both carriers at both locations. This justifies the Fermi model’s assertion that, near the Fermi level, electrons and holes contribute about equally to conductivity, regardless of which band contains the Fermi level.

**Figure 3:**
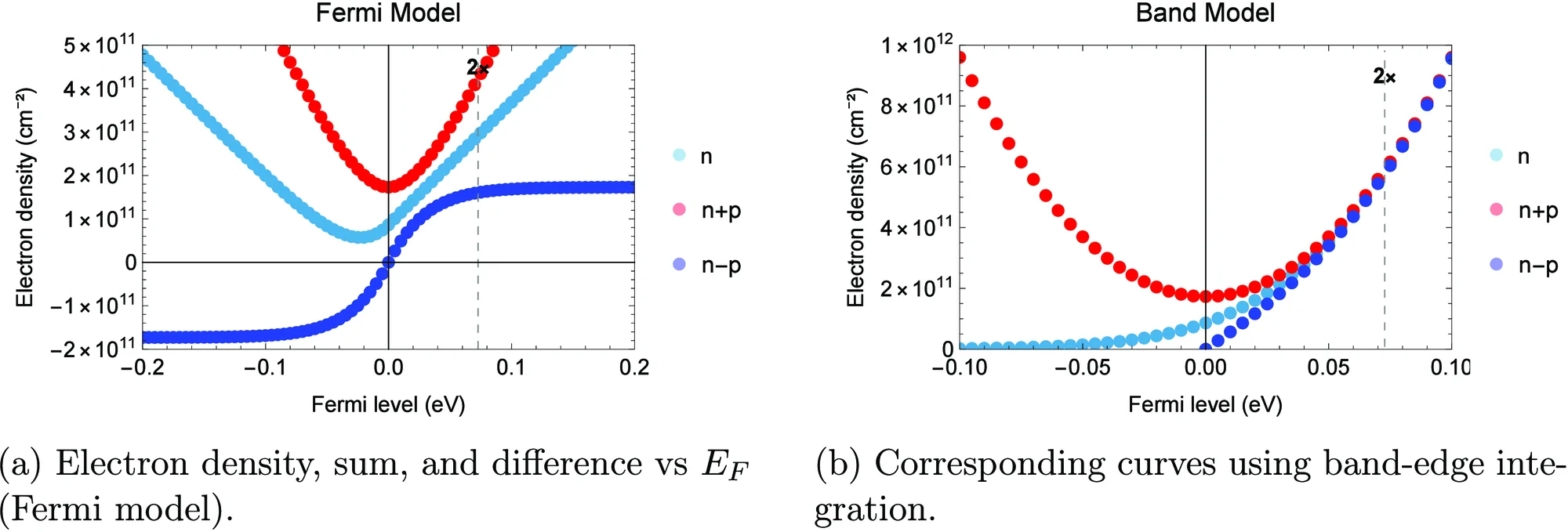
Carrier densities from Fermi-level vs band-edge counting. At *E*_F_ = 73 meV, the electron density from band-edge counting is overestimated by a factor of 2.

**Figure 4:**
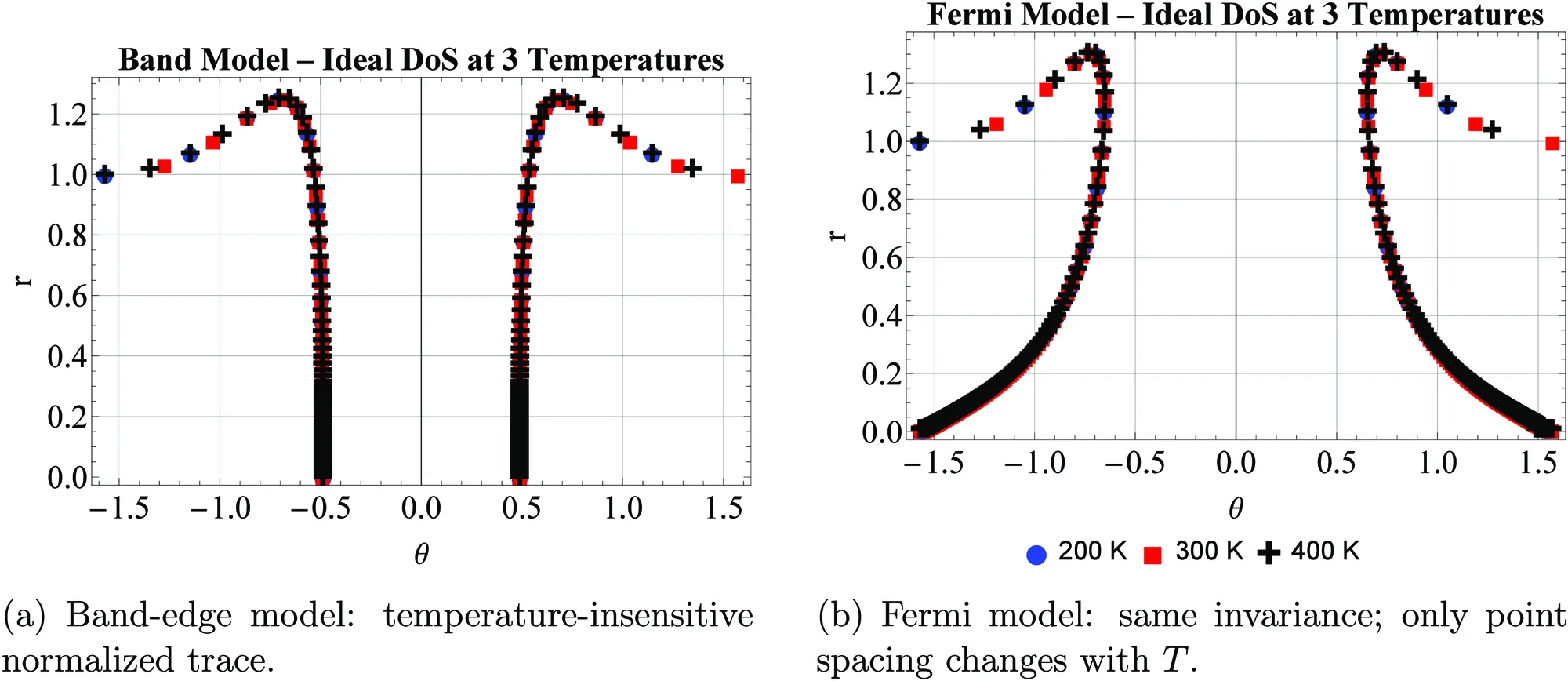
Temperature-insensitive normalized polar traces for graphene.

Moreover, the carrier density is highly sensitive to the local DoS at the Fermi level. The contributions of electrons and holes are dictated by the immediate DoS above and below the Fermi level, respectively. This explains the positive Hall effect of metals such as beryllium,^21^ where viewing holes as positive carriers is insufficient—genuine positive carriers do not exist (SI Section 5).^22^ For typical semimetals and degenerate semiconductors, the marginally greater electron contribution on the n-type side yields the expected negative Hall effect.

Independently of any interpretation, some occupied electronic states produce a positive Hall contribution while others produce the expected negative one; whether this is best described with holes or with electrons alone remains debated, and our use of p and n carries no commitment to holes. In practice, the Fermi model’s correction is most significant for Dirac and Weyl materials—including graphene,^23^ Weyl semimetals,^24^ and topological insulator surface states^25,26^ —where states share identical orbital character across the Dirac/Weyl point and the linear dispersion gives electrons and holes the same speed |*v*| = *v*_F_, leaving no band-edge feature to distinguish the two. For traditional semiconductors like Si or GaAs, the band model retains practical value: conduction and valence band carriers have different orbital characters and mobilities, and approximately isotropic bands near extrema make the uniform-drift assumption reasonable.

Table 1 quantifies the correction factors at representative doping levels, demonstrating that the discrepancy grows with increasing Fermi-level shift. These factors explain why nominally identical samples show order-of-magnitude mobility variations when measured at different doping levels. The applicability ranges follow directly: the two schemes’ integrals coincide exactly at neutrality and agree within ∼20% for |*E*_F_| ≲ *k*_B_ *T* (at 300 K, |*n*| ≲ 2 × 10^11^ cm^–2^), so band-edge extraction is adequate at that level only within about one thermal energy of the Dirac point, with the corrections of Table 1 applying beyond. We emphasize that this low-doping agreement reflects the convergence of the two integrals as *E*_F_ → 0 rather than a separate validation of band-edge counting—at all dopings the Hall coefficient is controlled by the asymmetry of the DoS about the Fermi level—and it also explains why the discrepancy has escaped notice: most published sweeps remain within the window where the two schemes differ by less than the experimental uncertainty—and, more fundamentally, because in extraction use the band model’s free quantities exceed the number of measured curves, so agreement was guaranteed and the model was never tested until the parameter-free comparison of Figure 1.

**Table 1:** Representative Mobility Corrections under the Fermi Model^a^

doping (eV)	band model *n* (cm^–2^)	Fermi model *n* (cm^–2^)	mobility factor
0.05	2 × 10^11^	8 × 10^10^	2.5×
0.10	8 × 10^11^	2.5 × 10^11^	3.2×
0.20	3 × 10^12^	7 × 10^11^	4.3×

a The carrier density ratio directly translates to mobility enhancement, revealing that reported mobilities systematically underestimate true values by factors of 2–5.

In summary, the Fermi model redefines the integration limits for carrier counting from the band edges to the Fermi level and, with them, the interpretation of Hall and resistance measurements in semimetals: electrons and holes coexist in comparable quantities near the Fermi level regardless of the band it lies in, the local DoS at *E*_F_ governs both carrier density and mobility, and the parameter-free normalized trajectories replace the single-carrier and band-edge approximations. Most immediately, our work implies that the highest-quality graphene samples may have intrinsic mobilities exceeding 300,000–500,000 cm^2^/V·s when properly analyzed—significantly higher than current record claims—necessitating reevaluation of mobility benchmarks across the 2D materials literature.

## Supplementary Material



## Notes

DFT-calculated DoS for graphene (Figure S1), six DoS variants for polar representation analysis including ideal linear, modified slopes, disorder-induced puddles, and band-gap cases (Figure S2), Hall coefficient calculations under both integration schemes (Figure S3), physical basis of electrical conduction and Hall effect with Fermi surface illustration (Figure S4), discussion of the carrier definition and comparison with cyclotron resonance measurements, and relationship of the Fermi model to Boltzmann transport theory, including the exact reduction to band-edge integration for gapped systems, the zero-temperature limits, and the non-normalized high-doping relationships of both schemes (Section 6) (PDF)
