# Supplementary material for: A New Model Employing Fermi-Level Integration Limits to Correct Systematic Errors in the Extraction of Semimetal Carrier Densities and Mobilities
Source: Nano Lett. 2026 Aug 21;26(36):12104–10. doi: 10.1021/acs.nanolett.6c02845 (PMC13587662; doi:10.1021/acs.nanolett.6c02845)
Supplement: Supplementary file 1 [file nl6c02845_si_001.pdf]

# Supporting Information for "A new model employing Fermi-level integration limits to correct systematic errors in the extraction of semimetal carrier densities and mobilities"

Y. W. Sun,[1,2,*] D. Holec,[3] O. Fenwick,[2] M. J. Coak,[1] D. G. Papageorgiou,[2] C. J. Humphreys,[2,†] and D. J. Dunstan[4,‡]

[1]*School of Physics and Astronomy, University of Birmingham, Birmingham B15 2TT, United Kingdom*

[2]*School of Engineering and Materials Science, Queen Mary University of London, London E1 4NS, United Kingdom*

[3]*Department of Materials Science, Montanuniversität Leoben, Leoben 8700, Austria*

[4]*School of Physical and Chemical Sciences, Queen Mary University of London, London E1 4NS, United Kingdom*

(Dated: August 14, 2026)

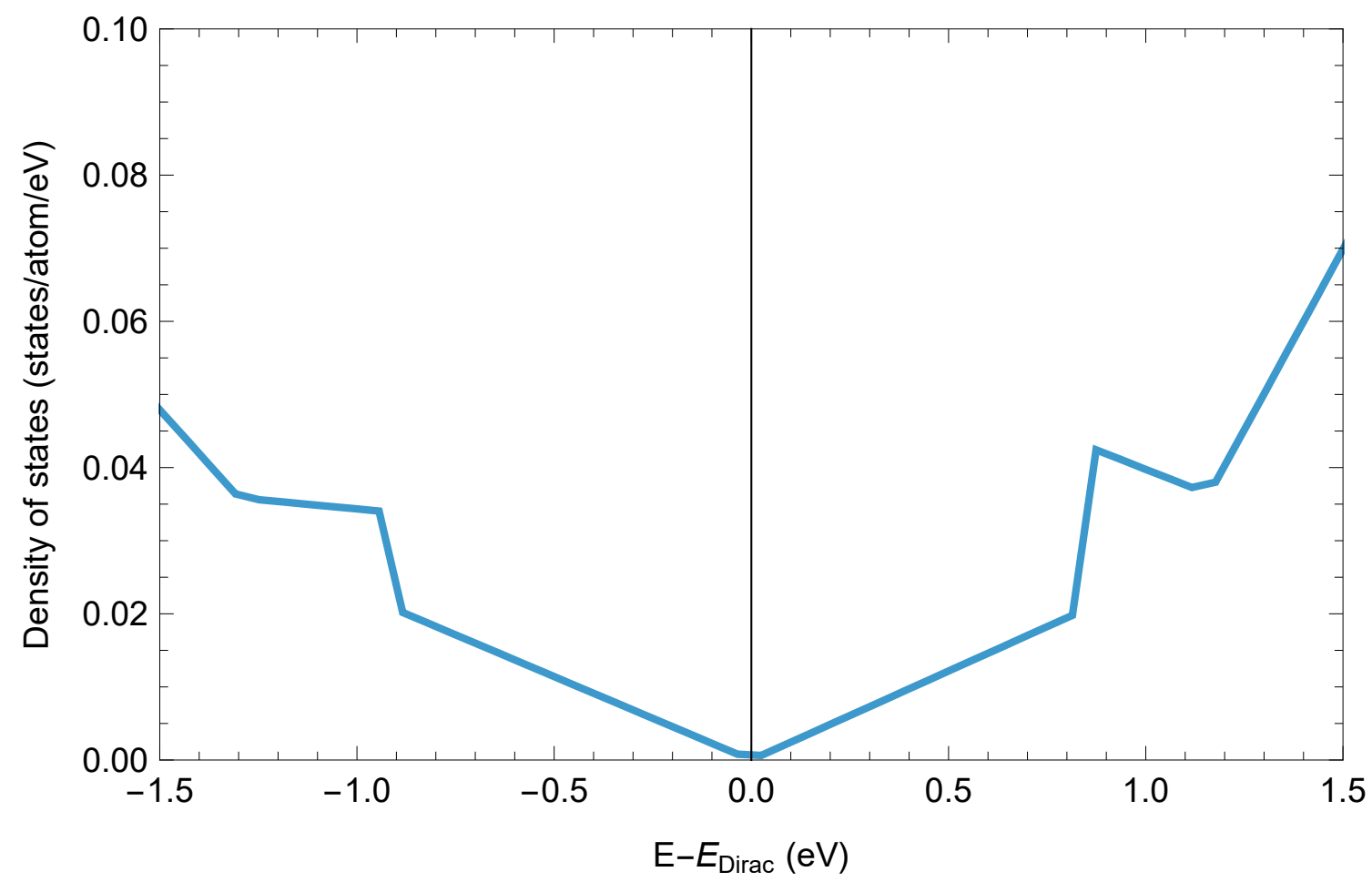


FIG. S1: Calculated DoS of free-standing graphene using DFT.

## LINEAR COEFFICIENT OF GRAPHENE DOS FROM DFT CALCULATIONS

We calculated using density functional theory (DFT) the Density of States (DoS) of free-standing monolayer graphene.[1] The DoS is shown in FIG. S1. Within the 0.8 eV doping range, the DoS of graphene is approximately linear on each side of the Dirac point, justifying the use of linear analytical DoS by many.[2] The calculated electron (hole) density of undoped graphene using the conventional band model is approximately $10^{11}$ $\mathrm{cm}^{-2}$, consistent with previous estimations.[3]

## DENSITY OF STATES VARIANTS USED IN THE POLAR REPRESENTATION ANALYSIS

To systematically investigate how the DoS affects the polar representation of transport properties under both the band-edge and Fermi-level integration schemes, we employed six different DoS models, as shown in FIG. S2. These variants were chosen to represent modifications that can occur in graphene and other semimetals due to various physical effects.

The six DoS models are:

1. **Ideal linear DoS**: The baseline model with a linear dispersion $D(E) = 0.0415|E|$ (states/atom/eV), representing pristine graphene with perfect Dirac cones, as in Fig. S1.

2. **Halved slope**: A reduced slope of $D(E) = 0.02|E|$.

3. **Doubled slope**: An increased slope of $D(E) = 0.08|E|$.

4. **Non-zero states (0.16 eV width)**: The ideal DoS modified with a constant DoS of 0.00332 states/atom/eV in the range $|E| < 0.08$ eV, modeling the effect of disorder-induced puddles or impurity states near the Dirac point.

5. **Non-zero states (0.08 eV width)**: Similar to model 4 but with a narrower energy range ($|E| < 0.04$ eV) and proportionally reduced constant DoS (0.00166 states/atom/eV).

6. **Band gap (0.16 eV)**: A true band gap of 0.16 eV width with zero states in the gap region ($|E| < 0.08$ eV), which can be induced in graphene through anisotropic strain.

As demonstrated in the main text (Figure 2), the band-edge integration model shows remarkable insensitivity to these DoS variations—all six variants produce nearly identical polar traces. In stark contrast, the Fermi-level integration model responds sensitively to DoS modifications: while slope changes (models 1-3) leave the polar trace largely unchanged, the introduction of non-zero states or a band gap (models 4-6) produces significant lateral shifts in the high-doping region of the polar plot. This fundamental difference in DoS sensitivity provides a crucial experimental test for distinguishing between the two carrier-counting schemes.

The choice of 0.16 eV for the gap width in model 6 is motivated by theoretical predictions for graphene on h-BN with specific crystallographic alignment, while the puddle models (4 and 5) represent the range of disorder effects commonly observed in supported graphene devices. These variants thus span the realistic range of DoS modifications encountered in experimental graphene systems.

## CALCULATED HALL COEFFICIENT WITH DOPING UNDER BOTH THE FERMI AND BAND MODELS

In FIG. S3 we plot the Hall coefficients calculated for graphene with the ideal DoS, at different Fermi levels, and their first derivatives, using the Fermi model (a), compared to the conventional band model (b). Closed-form high-doping relations quantify this comparison:

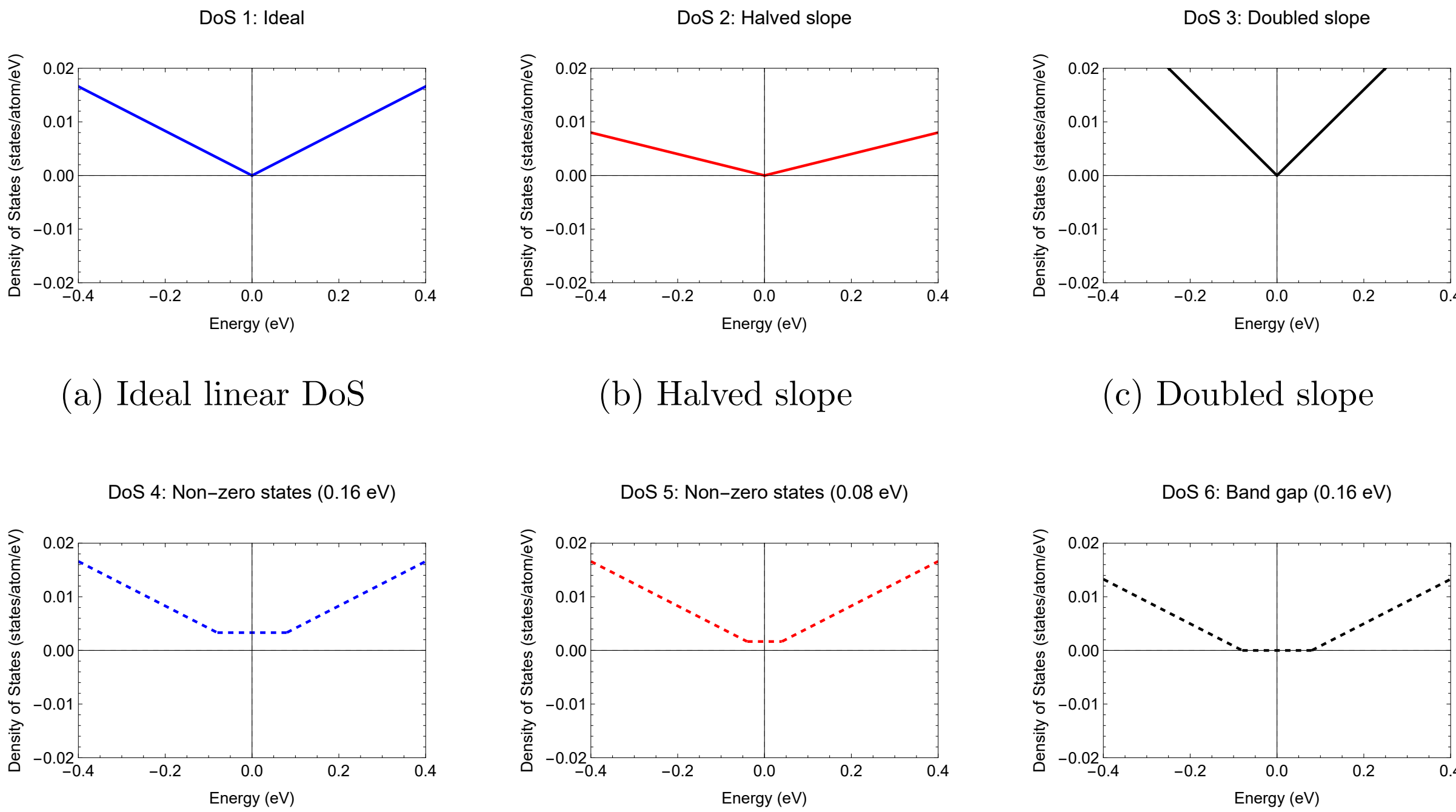


(a) Ideal linear DoS (b) Halved slope (c) Doubled slope

(d) Non-zero states (0.16 eV) (e) Non-zero states (0.08 eV) (f) Band gap (0.16 eV)

FIG. S2: Six density of states (DoS) variants used in the polar representation analysis. (a) Ideal linear DoS representing pristine graphene. (b) Halved slope DoS. (c) Doubled slope DoS. (d) Non-zero states at the Dirac point with 0.16 eV width, modeling disorder-induced puddles. (e) Non-zero states with 0.08 eV width. (f) True band gap of 0.16 eV, which can be induced under anisotropic strain. The vertical dashed lines at $E = 0$ mark the Dirac point (or gap center for variant f).

with the total induced charge $Q = cE_F^2/2$, $R_H \to -0.43/(eQ)$ under the Fermi model and $R_H \to -1/(eQ)$ under the band model, both $\propto 1/Q$; see the section "Relation of the Fermi model to Boltzmann transport theory" below.

## THE NATURE OF ELECTRICAL CONDUCTION AND THE HALL EFFECT AND THEIR RELATION TO CARRIER DENSITY

The electrical conduction of a material arises due to an external electric field exerting a force on electrons, as illustrated by the simplified model in FIG. S4. When an electric field is applied toward the left, electrons experience a force toward the right, causing the Fermi circle of a material (with its Fermi level in the conduction band) to rigidly shift toward the

(a)

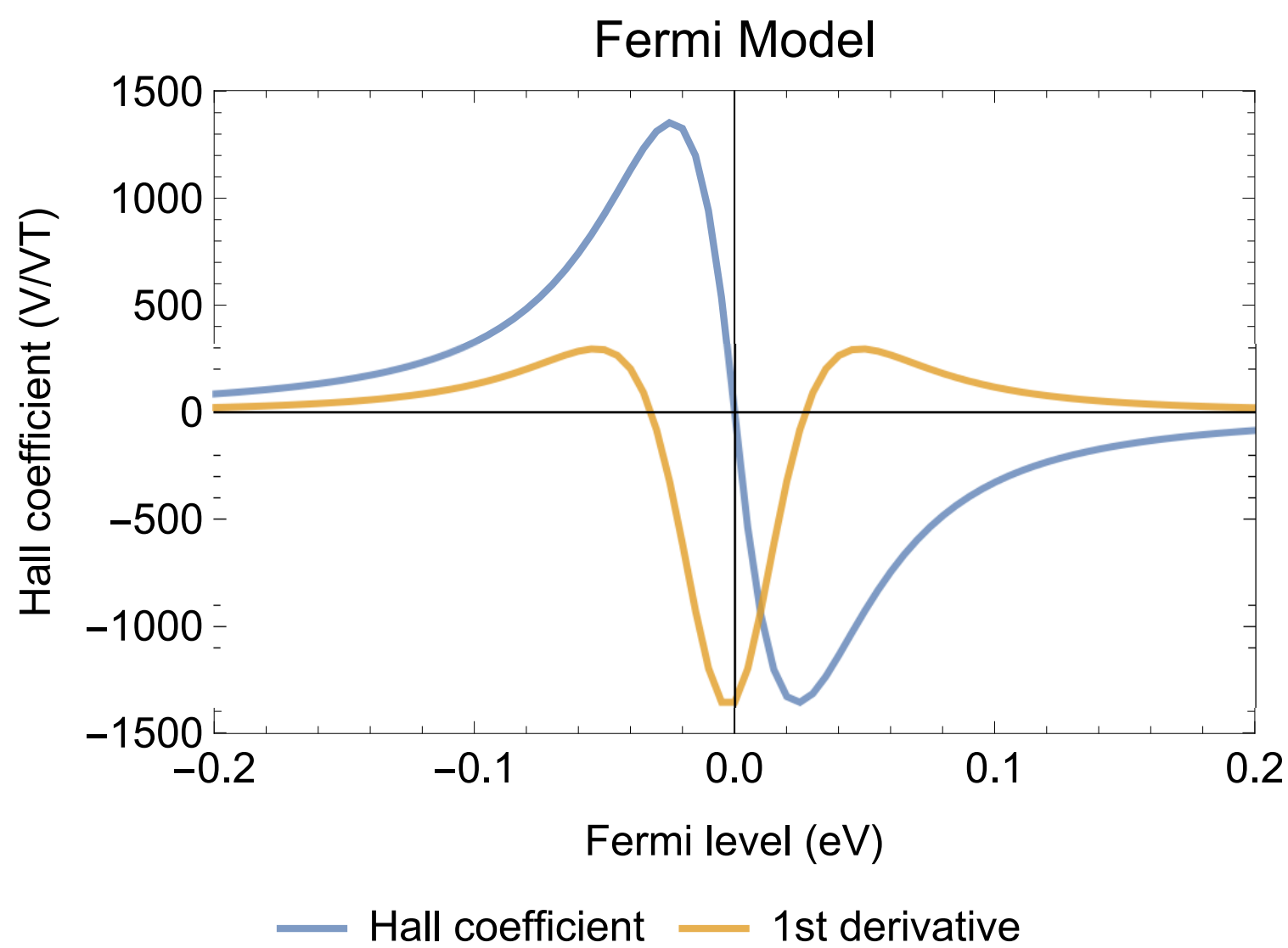


(b)

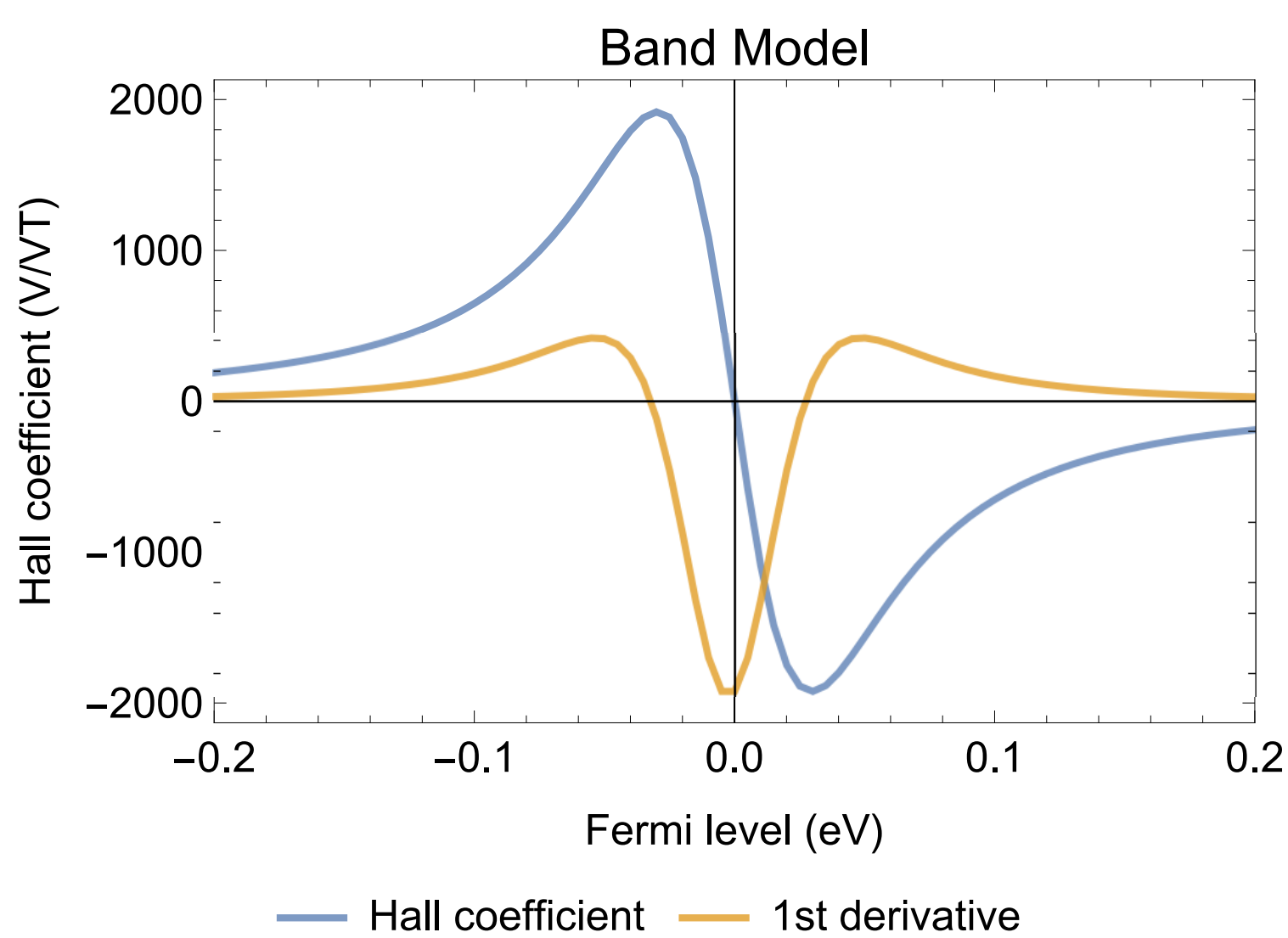


FIG. S3: (a) The calculated Hall coefficient and its first derivative as a function of the Fermi level for graphene with the ideal DoS following the Fermi model. (b) Similar calculation as in (a), but based on the conventional band model. Despite significant differences in carrier density between the two models, both predict comparable behavior for the Hall coefficient across varying doping levels.

right in two-dimensional $k$-space, resulting in current flow toward the left.

There are two mathematically equivalent but conceptually different ways to describe this conduction process: (1) all electrons within the entire conduction-band circle have a small drift velocity (typically on the order of cm/s) superposed on their large intrinsic velocities,

or (2) more physically accurately, only a small population of electrons near the Fermi surface make large changes (e.g. reversal) of their large intrinsic Fermi velocities (on the order of thousands of km/s). The second description is particularly important as it correctly accounts for velocity anisotropy when dealing with non-spherical Fermi surfaces.

For a p-type doped material, electrons in the valence band similarly experience a force to the right. However, conduction now occurs primarily due to electrons near the left edge of the Fermi circle moving into empty states on the right edge, effectively shifting the circle leftward. Three equivalent descriptions exist here: (1) a small number of electrons near the Fermi surface move rapidly from large negative $k_x$ to large positive $k_x$, (2) all empty states ("holes") shift left at a slow drift velocity, or (3) many electrons in the valence band move collectively leftward at slow drift velocity, with the critical caveat that their effective mass is negative, causing their shift to oppose the force direction.

Therefore, clarity about what precisely is meant by "carriers"—including their associated velocities, effective masses, and mobilities—is essential. In graphene, for example, the effective mass is commonly related directly to the Fermi velocity. Thus, carrier density and mobility values obtained through cyclotron resonance experiments (which measure effective masses linked directly to Fermi velocity) cannot be directly compared to values from conventional semiconductor measurements (Hall and resistivity methods), since they implicitly define "carriers" differently.

Under this accurate picture, a material's electrical conduction and Hall effect fundamentally reflect the rigid translation of its Fermi surface under external fields, which is highly sensitive to its local band structure and DOS near the Fermi level. When using traditional semiconductor integration limits (integration from the band edge rather than the Fermi level) to calculate carrier density for materials with Fermi levels within their bands (e.g., doped graphene), the approach fails to capture critical local DOS properties at the Fermi surface, leading to physically inaccurate carrier densities and mobilities. Hence, the semiconductor approach is insufficient for accurately describing graphene or similar semimetals.

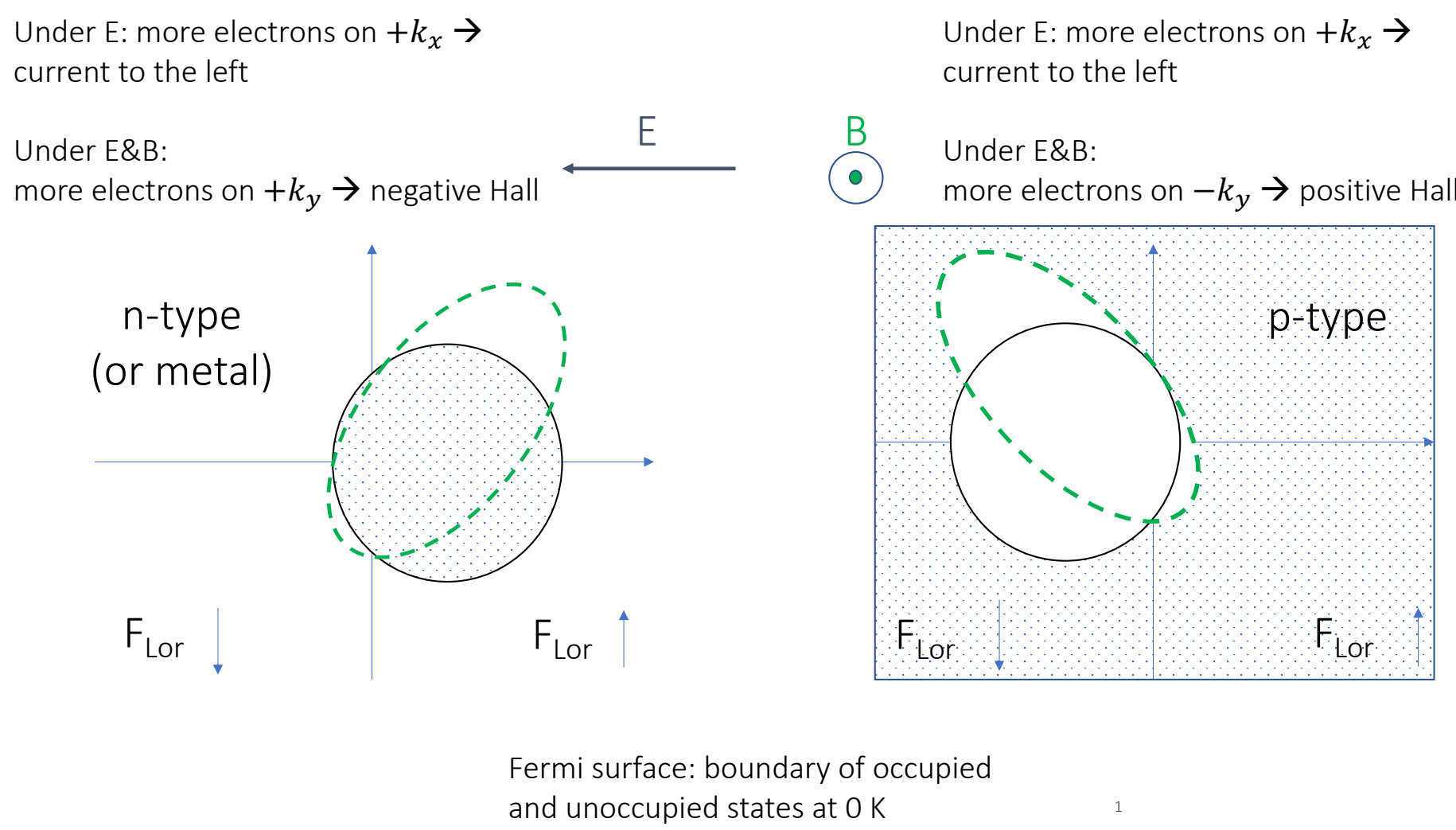


FIG. S4: Fermi circles in 2D $k$-space of materials with Fermi level in conduction band (left) and in valence band (right), under electric field (black solid circles), and under both electrical and magnetic field (green dashed ovals). The dots mark occupied states. The directions of Lorentz force on electrons with $+k$ and $-k$ under the magnetic field are labeled.

## PROBLEMS OF CONSIDERING HOLES AS POSITIVELY CHARGED PARTICLES

First, the concept of two carrier types (electrons and holes) cannot explain the positive Hall effect. In a Hall effect measurement, one has the source (drive) current along $+x$ direction, and the magnetic field along $+z$ direction. Lorentz force on electrons moving along $-x$ points towards $-y$. Similarly, holes (positive charge carriers, moving along $+x$) experience force toward $-y$. The sign of Hall voltage is then determined by this competition between electrons and holes. However, a positive charge carrier does not exist. Transport by holes in semiconductors is actually the movement of valence electrons in the opposite direction. Therefore, in a Hall measurement on a semiconductor, what really happens is that two types of electrons (valence and conduction) both move along $-x$, and are both pushed towards $-y$. Positive Hall effect, routinely observed in metals and p-type doped semiconductors, would not happen. Fig. S4 illustrates the mechanism that enables a positive Hall effect. With an electric field applied along $-x$ and a magnetic field along $+z$ direction,

for both n- and p-type doped materials, Lorentz force on the electrons with $-k_x$ is along $-y$ and on those with $+k_x$ is along $+y$. The magnitude of the Lorentz force on an electron is proportional to $k_x$. The sign of the Hall effect is determined by comparing the populations of electrons at positive and negative $k_y$ values — no need to introduce the concept of holes. That an n-type doped material has negative Hall effect is straightforward: Fermi circle shifts to $+k_x$; the majority of the circle on $+k_x$ is distorted upwards and the minor part on $-k_x$ downwards; there are more electrons with $+k_y$ in the green Fermi oval. For a p-type doped material, after Fermi circle of empty states drifts to $-x$ under the electric field along $-x$, electrons near the top edge of the Fermi circle on the $-k_x$ side are pushed down and accumulate at the bottom of the empty Fermi circle, distorting it to $+k_y$. The minor part on the $-k_x$ side is distorted to $-k_y$. Overall, there are more empty states with $+k_y$ in the green Fermi oval (hence more electrons with $-k_y$), resulting in a positive Hall effect. Similarly to the 1D conduction behavior, the drift of Fermi surfaces is the determining factor also in the 'Hall behavior' (indicated by Hall coefficient), and therefore, applying a single-carrier approximation based on carrier densities calculated from band edges—thus insensitive to the local DoS at the Fermi level—is questionable.

## RELATION OF THE FERMI MODEL TO BOLTZMANN TRANSPORT THEORY

The carrier densities of the Fermi model are weighted state counts under the transport kernel of Boltzmann theory. Integrating by parts, with $N(E) = \int_{E_F}^{E} D(E')\,dE'$ and $M(E) = \int_{E}^{E_F} D(E')\,dE'$ (both vanishing at $E = E_F$, and the boundary terms at $\pm\infty$ vanishing by the exponential decay of $f$ and $1-f$),

$$n = \int_{E_F}^{\infty} Df\,dE = \int_{E_F}^{\infty}\Big(-\frac{\partial f}{\partial E}\Big)N(E)\,dE, \qquad p = \int_{-\infty}^{E_F} D(1-f)\,dE = \int_{-\infty}^{E_F}\Big(-\frac{\partial f}{\partial E}\Big)M(E)\,dE. \tag{1}$$

The kernel $-\partial f/\partial E$ is the weight that determines the dc response in the linearized Boltzmann equation; the Fermi level is its only characteristic energy, and it is therefore the counting reference of the scheme. Four consequences follow.

*(i) Exact reduction in the gapped, non-degenerate limit.* If $E_F$ lies in a gap, $D(E) = 0$ between $E_V$ and $E_C$, so $\int_{E_F}^{\infty} Df\,dE = \int_{E_C}^{\infty} Df\,dE$ and $\int_{-\infty}^{E_F} D(1-f)\,dE = \int_{-\infty}^{E_V} D(1-f)\,dE$ identically. All standard semiconductor results are contained unchanged; the schemes differ only when occupied states extend continuously below $E_F$. This reduction is directly

visible in Figure 2(b) of the main text: while $E_F$ lies within the gap the two prescriptions coincide, so the gap-variant Fermi trace follows the (DoS-insensitive) band-model line—an intrinsic-regime ray with $\tan\theta \to 1/2$ for constant equal mobilities under the trace's own normalization—and departs from it only where $E_F$ crosses the band edge. The turning point of the gap-variant trace is precisely this crossing; its sharpness reflects the step edge of DoS variant 6 relative to $k_BT$, and is rounded for a density of states rising continuously from zero at the edge.

*(ii) Zero-temperature limit and non-normalized relations.* For a linear DoS $D(E) = c|E|$ and $E_F \gg k_BT$,

$$n, p = cE_F k_B T \ln 2 \pm \frac{\pi^2}{12} c\,(k_BT)^2, \qquad n + p = 2\ln 2\, D(E_F)\, k_BT, \qquad n - p = \frac{\pi^2}{6} c\,(k_BT)^2, \tag{2}$$

so that the $(k_BT)^2$ factors cancel exactly in the Hall coefficient,

$$R_H = \frac{p-n}{e(p+n)^2} \;\longrightarrow\; -\frac{\pi^2}{24\ln^2 2}\frac{1}{e\,c\,E_F^2}, \tag{3}$$

a finite, temperature-independent limit (verified numerically to five significant figures between 75 and 300 K). Since $cE_F^2/2$ is the total induced charge $Q$, this reads $R_H \to -0.43/(eQ)$, while band-edge integration gives $R_H \to -1/(eQ)$: both schemes predict $R_H \propto 1/Q$, so the routinely verified linearity of the Hall response with gate charge is common to both, and their entire non-normalized difference is a doping-independent prefactor of 2.3—within the Hall-scattering-factor uncertainty in graphene ($s \approx 0.5$–4 [main text Ref. 14]), which the double normalization cancels. The mobility extracted at temperature $T$, $\mu = \sigma/[e(n+p)]$, correspondingly scales as $v_F^2\tau/k_BT$; using the Boltzmann conductivity $\sigma = \frac{1}{2}e^2v_F^2\tau D(E_F)$ gives $e(n+p)\mu = \sigma$ identically, with every intermediate quantity finite at each temperature. The vanishing of $n+p$ as $T \to 0$, compensated by the growth of the per-carrier response, is the standard degenerate-conductor statement that the current is carried by a small population near the Fermi surface moving at $v_F$ rather than by all carriers drifting slowly (Section 4).

*(iii) Scope of the single-band equivalence.* For the scalar conductivity of a single isotropic band, counting all occupied states with a drift mobility and counting the Fermi-window population with the per-carrier response above are mathematically equivalent, both yielding $\sigma = \frac{1}{2}e^2v_F^2\tau D(E_F)$ in two dimensions. The equivalence does not extend to the pair $(R_H, R_{xx})$ of a gapless conductor: $R_{xx}$ depends only on the sum of the two channels while

$R_H$ depends on their partition, and it is on the joint normalized trajectory—where the two counting schemes make geometrically distinct, parameter-free predictions—that experiment discriminates between them (main text, Figure 1). In conventional use, by contrast, the band-edge scheme operates as an extraction tool with the mobility and its dependences as per-device free functions; on the un-normalized observables its adjustable quantities exceed the measured curves and it cannot be falsified. The normalization removes these quantities and restores falsifiability to both schemes.

*(iv) Domain of validity.* The zero-temperature expressions above establish the internal consistency of the scheme; they are not a claim that semiclassical two-carrier extraction—under either counting scheme—remains the appropriate physical description at arbitrarily low temperature. As $T \to 0$, quantum corrections to the conductivity (weak localization, electron–electron interactions) and, at finite field, Landau quantization dominate the magnetotransport, and the Hall response is described by the quantum Hall effect, in which both schemes count the same total charge through identical plateau filling factors. The explicit temperature-sweep calculations in this work are performed between 200 and 400 K, within the semiclassical domain.

---


* yiwei.sun@qmul.ac.uk

† c.humphreys@qmul.ac.uk

‡ d.dunstan@qmul.ac.uk



[1] G. Kresse and J. Furthmüller, Efficient iterative schemes for ab initio total-energy calculations using a plane-wave basis set, Physical Review B **54**, 11169 (1996).

[2] A. H. Castro Neto, F. Guinea, N. M. R. Peres, K. S. Novoselov, and A. K. Geim, The electronic properties of graphene, Reviews of Modern Physics **81**, 109 (2009).

[3] K. S. Novoselov, A. K. Geim, S. V. Morozov, D. Jiang, Y. Zhang, S. V. Dubonos, I. V. Grigorieva, and A. A. Firsov, Two-dimensional gas of massless dirac fermions in graphene, Nature **438**, 197 (2005).
